# Viral infection and glioma: a meta-analysis of prognosis

**DOI:** 10.1186/s12885-020-06796-3

**Published:** 2020-06-12

**Authors:** Zehao Cai, Shoubo Yang, Xiaoyan Li, Feng Chen, Wenbin Li

**Affiliations:** Department of neurosurgery, Beijing Tiantan Hospital, Capital Medical University, No.119 West Nansihuan Road, Beijing, 100071 China

**Keywords:** viral infection, glioma, prognosis, detection, PCR, immunohistochemistry

## Abstract

**Background:**

Glioma is the most common primary brain tumor, occurring due to the carcinogenesis of glial cells in the brain and spinal cord. Many aspects of the mechanism of its tumorigenesis remain unknown. The relationship between viral infection and glioma is one of the most important research aspects in this field. Currently, there is a lack of systematic reviews and meta-analyses to evaluate the effect of viral infection on the prognosis of glioma patients. The purpose of this study was to evaluate the relationship between viral infection and the prognosis of glioma patients, aimed at evaluating the prognostic value of the detection of viral infection.

**Methods:**

Through careful and comprehensive retrieval of results from the PubMed, Embase, and Cochrane databases, eligible articles were selected strictly according to the inclusion and exclusion criteria. The regional sources, detection methods, detection indicators, patient survival, and other data from the samples in the papers were extracted, and the integrated analysis was conducted using Stata 15.1. We conducted a subgroup analysis of the relationship between the degree of infection and prognosis in cytomegalovirus (CMV) patients.

**Results:**

A total of 11 studies were included in the analysis. Among them, 7 studies involved the relationship between CMV infection and the prognosis of patients with glioma, 2 studies involved human papillomavirus (HPV), 2 studies involved human herpesvirus-6 (HHV-6), and one study involved simian virus 40 (SV40), woolly monkey sarcoma virus (WMSV) and human endogenous retrovirus K113 (HERV-K113). In the CMV study, the pooled Hazard ratio (HR) of Overall survival (OS) was 1.024 (CI: 0.698–1.501), with a P value of 0.905. The pooled HR of Progression free survival (PFS) was 1.067 (CI: 0.770–1.478), with a P value of 0.697. The pooled HR value of low-degree infection versus high-degree infection was 1.476 (CI: 0.799–2.727), with a P value of 0.213. In the HPV study, the pooled HR of OS was 1.467 (CI: 0.552–3.901), with a P value of 0.443.

**Conclusion:**

CMV infection has no significant effect on the prognosis of glioma patients. Using the IEA as the detection index, the degree of CMV infection was found to have a significant impact on the prognosis of glioma patients; it was not found to possess a significant prognostic value after the integration of different indicators. Neither HPV nor HHV-6 infection has a significant effect on the prognosis of glioma patients. SV40 and WMSV infection are associated with poor prognosis in patients with low-grade glioma.

**Trial Registration:**

This meta-analysis registered in https://www.crd.york.ac.uk/PROSPERO/, PROSPERO ID: CRD42019127648.

## Background

Glioma is the main type of brain tumor, with a poor prognosis and quality of life. Despite the standard treatment regimen of surgery, postradiotherapy, concurrent chemotherapy, and adjuvant chemotherapy, the survival of glioblastoma patients is still only about 15 months. Cytomegaloviruses are a member of the family Herpesviridae. It is named “cytomegalovirus” because the infected cells are swollen and have large nuclear inclusions. Human herpesvirus-6 (HHV-6) has the morphological characteristics of typical simplex viruses and is similar to CMV in that it mainly infects CD4+T cells, CD8+T cells, mononuclear macrophages, and NK cells. Human papillomavirus (HPV) is a type of papovavirus belonging to the Papovaviridae family. It is a spherical DNA virus and can cause the proliferation of the squamous epithelia of the human skin mucosa. Simian virus 40 (SV40) is an oncogenic virus found in both humans and monkeys; its genome is 5.2 KB in size and comprises circular double-stranded DNA. The virus has a diameter of 45 nm, a maturation site at the nucleus, and no envelope.

## Methods

### Search strategy

We carefully searched for literature published until February 28, 2019 in the PubMed, Embase, and Cochrane databases. The search terms mainly included the following terms: "Glioma", "Viruses", "RNA Virus Diseases", "DNA Virus Diseases", and "Central Nervous System Viral Diseases". The search strategy is adjusted according to documents from different databases; the search strategy of PubMed is: ((((((((((((((((("Glioma"[Mesh]) OR Gliomas[Title/Abstract]) OR Glial Cell Tumors[Title/Abstract]) OR Glial Cell Tumor[Title/Abstract]) OR Tumor, Glial Cell[Title/Abstract]) OR Tumors, Glial Cell[Title/Abstract]) OR Mixed Glioma[Title/Abstract]) OR Glioma, Mixed[Title/Abstract]) OR Gliomas, Mixed[Title/Abstract]) OR Mixed Gliomas[Title/Abstract]) OR Malignant Glioma[Title/Abstract]) OR Glioma, Malignant[Title/Abstract]) OR Gliomas, Malignant[Title/Abstract]) OR Malignant Gliomas[Title/Abstract])) AND (((((((((("Viruses"[Mesh]) OR virus infection[Title/Abstract]) OR Viral Gene Expression[Title/Abstract]) OR Gene Expression, Viral[Title/Abstract]) OR Viral Expression[Title/Abstract]) OR Expression, Viral[Title/Abstract])) OR (((((("RNA Virus Infections"[Mesh]) OR Infections, RNA Virus[Title/Abstract]) OR Infection, RNA Virus[Title/Abstract]) OR RNA Virus Infection[Title/Abstract]) OR Virus Infection, RNA[Title/Abstract]) OR Virus Infections, RNA[Title/Abstract])) OR (((((("DNA Virus Infections"[Mesh]) OR Infections, DNA Virus[Title/Abstract]) OR DNA Virus Infection[Title/Abstract]) OR Infection, DNA Virus[Title/Abstract]) OR Virus Infection, DNA[Title/Abstract]) OR Virus Infections, DNA[Title/Abstract])) OR (((((((((((("Central Nervous System Viral Diseases"[Mesh]) OR Viral Infections, Central Nervous System[Title/Abstract]) OR Infections, CNS, Viral[Title/Abstract]) OR Infections, Viral CNS[Title/Abstract]) OR Infections, Viral CNS[Title/Abstract]) OR CNS Infection, Viral[Title/Abstract]) OR CNS Infections, Viral[Title/Abstract]) OR Infection, Viral CNS[Title/Abstract]) OR Viral CNS Infection[Title/Abstract]) OR Viral CNS Infections[Title/Abstract]) OR Viral Diseases, and Central Nervous System[Title/Abstract]) OR Central Nervous System Viral Infections[Title/Abstract]))).

### Eligibility criteria and quality assessment

Literatures that met the following criteria were included in the study: (1) the samples were brain tumor tissue samples from clinical patients, with clear source records and clear pathological diagnosis, (2) studies with clear specimen detection methods and indicators, (3) studies exploring the correlation between the expression of the virus in glioma cells and the survival outcome of patients, and (4) HR or relative risk (RR), 95% CI or SE, and Kaplan-Meier curves were reported in the studies. Otherwise, we contacted the corresponding author to obtain the required data.

Literatures that met the following criteria were excluded: (1) the written language was not English, (2) reviews, letters, editorials, and meeting records, (3) study focusing on gene expression, (4) the detection samples were not from the glioma samples of the patients, (5) neither the viral antigen nor DNA were detected, and (6) sample cases were from a database.

Literatures included in the study were evaluated according to the guidelines of the observational studies in the epidemiology group (MOOSE) (Stroup et al. 2000)[1, 2]. The main assessment items are: (1) clear source of specimens, (2) rigorous and transparent experimental design, (3) a clear definition of positive viral infection, and (4) long enough survival follow-up.

### Data extraction

iResearchers extract the following research data through the standard form: (1) first author's name and year of publication, (2) regional origin of the study population and tumor classification, (3) detection method and detection index, (4) total number of subjects, and numbers of positive and control subjects, and (5) survival analysis by statistical methods, HR, 95% CI, SE, and *P* values. In the absence of survival data in the literature, we adopted the following strategies to extract the data: (1) download the patient information table, adopt the covariate indicators described in the literature, and use SPSS 22.0 to calculate the HR, 95% CI, and P values, (2) according to the extraction strategy presented by Jayne f. Tierney et al. [3], HR and 95% CI were extracted from Kaplan-Meier curves by using Photoshop Portable, Engauge Digitizer 10.8, and Excel 2013, and *P* values were calculated using Revman 5.3, (3) when it was difficult to extract the HR data, we recorded the OR values for 18 months of survival or the median survival time, and (4) obtaining the required data by contacting the corresponding author.

### Statistical analysis

Researchers used I2 statistics to evaluate the heterogeneity among different studies. If the I2 was < 50%, the fixed effect model was used. If the I2 was > 50%, the random effect model was used. The statistical software Stata 15.1 was used for calculating the I2 statistics. The final pooled effect size was calculated in case of the following: (1) HR, (2) 95% CI, (3) SE (HR), and (4) *P* value. An HR value of > 1 indicates that the dependent variable of the test group has a negative effect on the prognosis; an HR value < 1 indicates that the dependent variable of the test group has a positive effect on the prognosis. *P* < 0.05 indicates that the conclusion was considered statistically significant. The results were subjected to sensitivity analysis; the calculation model was selected according to the I2 value. If the effect value deviates greatly from the pooled value or exceeds the 95% CI range of the pooled value, it is considered that the study has a significant impact on the pooled result.

## Results

### Study characteristics

**Fig. 1:**
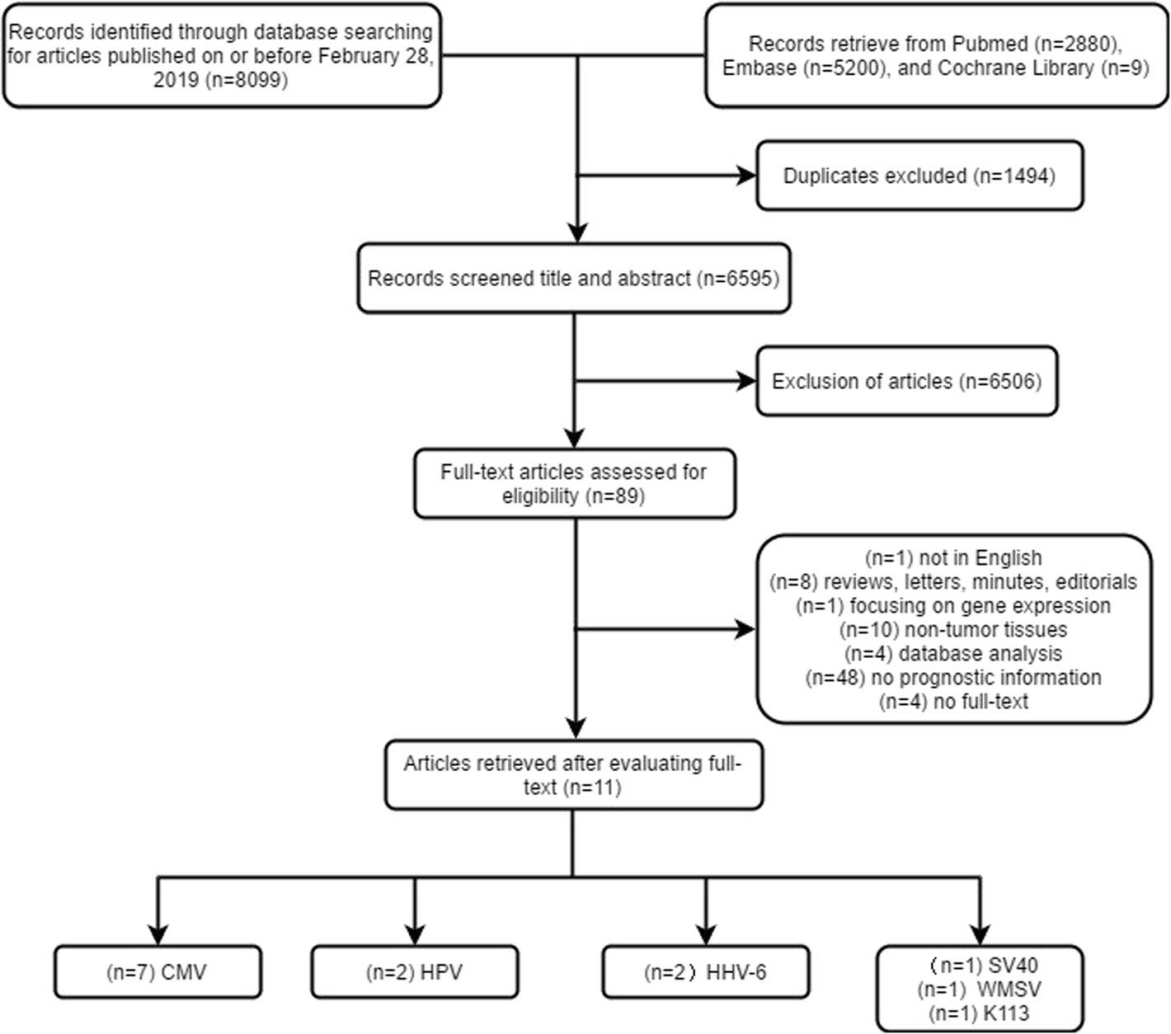
Flow diagram representing the selection process

A total of 8,089 records were searched, including 2,880 PubMed records, 5,200 Embase records, and 9 Cochrane records. Among them, 1,494 duplicates were excluded. After reading the titles and abstracts, 6,506 records were excluded. The researcher evaluated 85 full-text literatures and excluded 74 studies according to the exclusion criteria as follows: 1 study was not in English; 8 literatures in total were reviews, letters, minutes, and editorials; 1 study focused on gene expression; the samples in 10 studies were non-tumor tissues; research data from 4 studies were obtained from databases; and 48 studies had test data but no prognostic information. In the end, 11 studies were included in the analysis. Moreover, all these literatures were judged as qualified in the quality evaluation. The selection flow chart of this study is shown in Fig. 1.

### Infected virus

In this meta-analysis, several viruses were searched. In total, 7 studies involved CMV: (Han et al. 2018) [4], (Yang et al. 2017) [5], (Bahador et al. 2017) [6], (Ding et al. 2014) [7], (Rahbar et al. 2013) [8], and (Rahbar, a. et al. 2012) [9] ; 2 studies involved HPV: (Adnan Ali et al. 2019 )[10], (Vidone et al. 2014) [11] and (Wang et al. 2017) [12]; 2 studies involved HHV-6: (Crawford et al. 2009) [13] and (Crawford et al. 2009) [14]; and 1 study involved SV40, WMSV and HERV-K113: (Wang et al. 2017) [12]. Among these studies, there were differences between different detection methods and indicators (immunohistochemistry: IE1-72, PP65, LA; PCR: UL55). The data information for each study is shown in Table 1.

**Table 1:** Study information included in the meta-analysis

study	year	region	virus	grade	method	mark	purpose	N	positive	negative	M or U	comparison	effect size	HR	LL	UL	P value
**Han,S**	2018	China	CMV	GBM	IF	gB	OS	68	33	35	U	P VS N	HR	1	0.11	9.22	0.995
					IF	gH	OS	68	29	39	U	P VS N	HR	0.73	0.08	6.47	0.7689
**Yang, C. F.**	2017	China		GBM	PCR	UL73	OS	116	9	107	M	P VS N	HR	1.661	0.771	3.575	0.195
							PFS	116	9	107	M	P VS N	HR	1.761	0.86	3.603	0.122
**Bahador, M.**	2017	Norway		GBM	IHC	IE1	OS	172	65	107	M	P VS N	HR	0.845	0.53	1.35	0.48
**Ding, D.**	2014	China		glioma	IHC	IE1-72	PFS	67	51	16	M	P VS N	HR	1.068	0.518	2.2	0.859
					IHC	PP65	PFS	67	44	23	M	P VS N	HR	0.953	0.51	1.78	0.88
					PCR	UL55	PFS	67	35	32	M	P VS N	HR	0.847	0.475	1.511	0.574
**Stragliotto, G.**	2013	Sweden		GBM			OS				M	H VS L	HR	6.61	1.36	32.1	0.0192
**Rahbar, A.**	2013	Sweden		GBM	IHC	IEA	OS	75	11	64	M	H VS L	HR	2.2	1	4.38	0.036
							PFS					H VS L	HR	1.778	0.8902	3.5512	0.103
					IHC	LA	OS	75	32	43	M	H VS L	HR	1.14	0.7	1.86	0.6
							PFS					H VS L	HR	1.487	0.8933	2.4752	0.127
**Rahbar,A.**	2012	Sweden		GBM	IHC	IEA	OS>18 months	80	19	61	M	H VS L	Odds	6.604	1.359	32.094	0.019
						LA	OS>18 months	80	19	61	M	H VS L	Odds	3.758	0.425	3.758	0.67
**Adnan Ali, S. M.**	2019		HPV	GBM	PCR	GP5、6	OS	112	31	81	M	P VS N	HR	0.913	0.407	2.047	0.825
**Vidone, M.**	2014			GBM	PCR	MY/GP	OS	52	12	40	M	P VS N	HR	2.48	0.99	6.24	0.45
**Wang, Z.**	2017	China	SV40	LGG	PCR	A	OS	172	40	132	M	P VS N	HR	2.935	1.4894	5.7837	0.0019
			WMSV	LGG	PCR	B	OS	172	25	147	M	P VS N	HR	2.109	1.0662	4.1715	0.032
			CMV	glioma	PCR		OS	138	8	130	U	P VS N	HR	2.88	0.17	48.7906	0.4638
			K113	glioma	PCR		OS	308	28	280	U	P VS N	HR				0.6216
**Crawford, J. R.**	2009	USA	HHV-6	glioma	IHC	gp116	OS	173	65	108	U	P VS N	HR	1.07	0.76	1.5	0.6983
					IHC	p41	OS	167	53	114	U	P VS N	HR	1.25	0.8	1.95	0.3271
**Crawford, J. R.**	2009	USA	HHV-6	glioma	IHC	Gp116/64/54	OS	130	42	88	M	P VS N	median survival time	3.08 vs 3.25	years	0.245	
							PFS	130	42	88	M	P VS N	medain PFS	2.67 vs 2.25	years	0.653	

### Pooled data

#### CMV

**Fig. 2:**
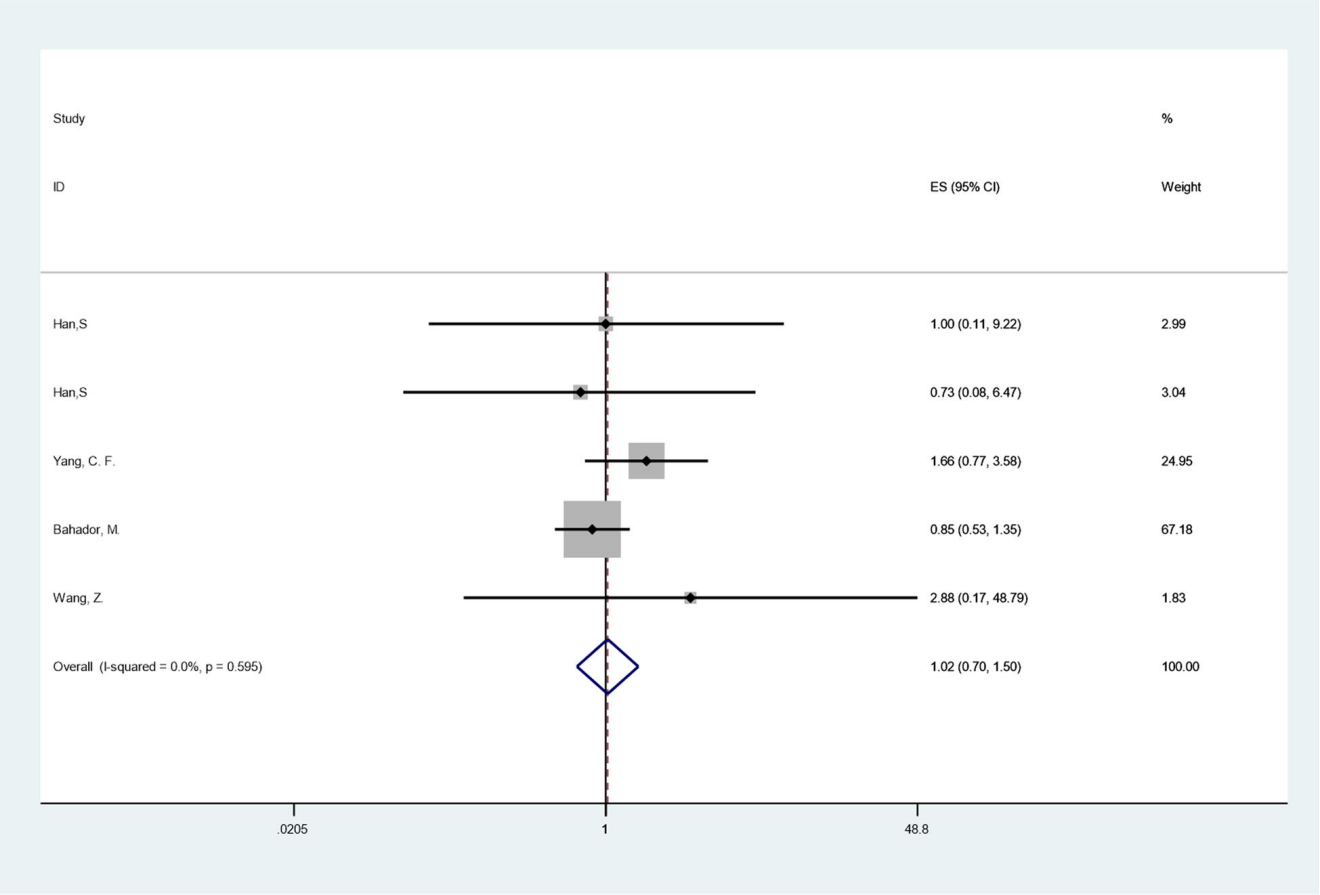
Pooled HR(OS) of CMV infection positive versus negative

In the subgroup analysis of OS, the heterogeneity test result of I2 was 0.0% (< 50%); thus, the fixed effect model was selected, and the final pooled HR was 1.024 (CI: 0.698–1.501), and the P value was 0.905, which was not statistically significant. Infection of glioma cells with CMV was not considered to have a significant impact on the patients' OS. Sensitivity analysis showed that individual studies or indicators had no significant influence on the combined results. The results of the combination of individual indicators for each study are shown in Fig. 2.

**Fig. 3:**
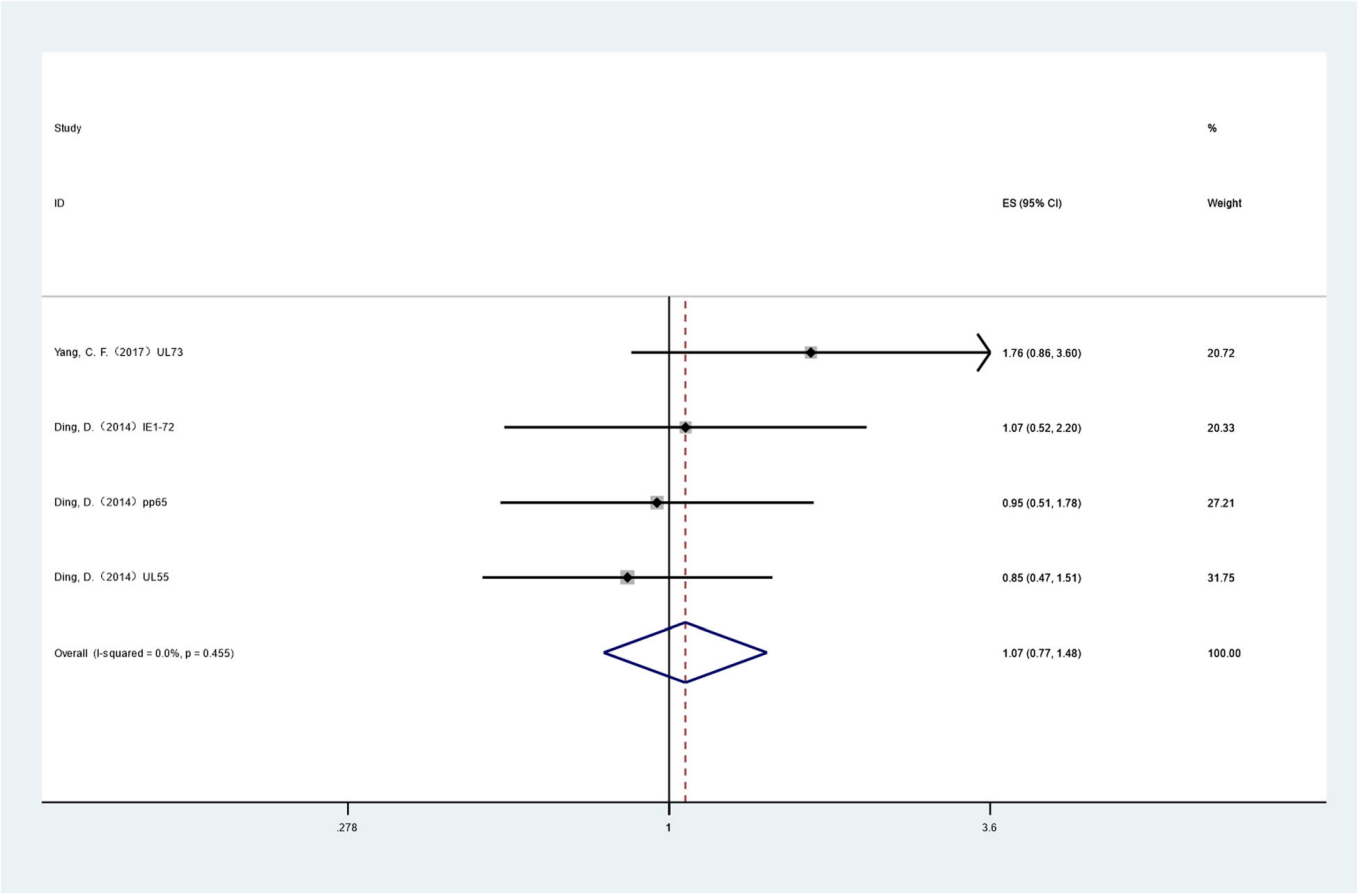
Pooled HR(OS) of CMV infection positive versus negative

In the subgroup analysis of FPS, the heterogeneity test result of I2 was 0.0% (< 50%); thus, the fixed effect model was selected, and the final pooled HR was 1.067 (CI: 0.770–1.478), and the P value was 0.697, which was not statistically significant. No significant effect of CMV infection on the patients' PFS was considered. Sensitivity analysis showed that single studies had no significant influence on the combined results. The results of the combination of individual indicators for each study are shown in Fig. 3.

In a study on tumor infection degree and patient prognosis (Rahbar et al. 2013), the HR of HCMV-IEA for patients with high levels of infection versus those with low levels was 2.146 (CI: 1.0515–4.3796), with a P value of 0.0359, which was statistically significant. However, there was no significant difference in the results of HCMV-LA detection in the same population (P= 0.606). Compared with the two trials with different indicators in this study, the I2 value was 52.2% (> 50%); thus, the random effect model was adopted. The pooled HR value of low-grade infection was 1.476 (CI: 0.799–2.727), and the P value was 0.213, showing no statistical significance. Indicator selection plays an important role in evaluating the relationship between viral infection and prognosis. In another study by Rahbar et al. (2012), we extracted OR values for the survival period of 18 months. When the IEA was used as the indicator, the OR value was 6.604 (CI: 1.359–32.094) and the P value was 0.019, showing no statistical significance. When LA was used as the index, the OR value was 3.758 (CI: 0.425–3.758) and the P value was 0.67, showing no statistical significance. In the above two studies, the degree of CMV infection showed significant prognostic value when the IEA was used as the monitoring index. LA was not observed to have the same prognostic effect.

#### HPV

**Fig. 4:**
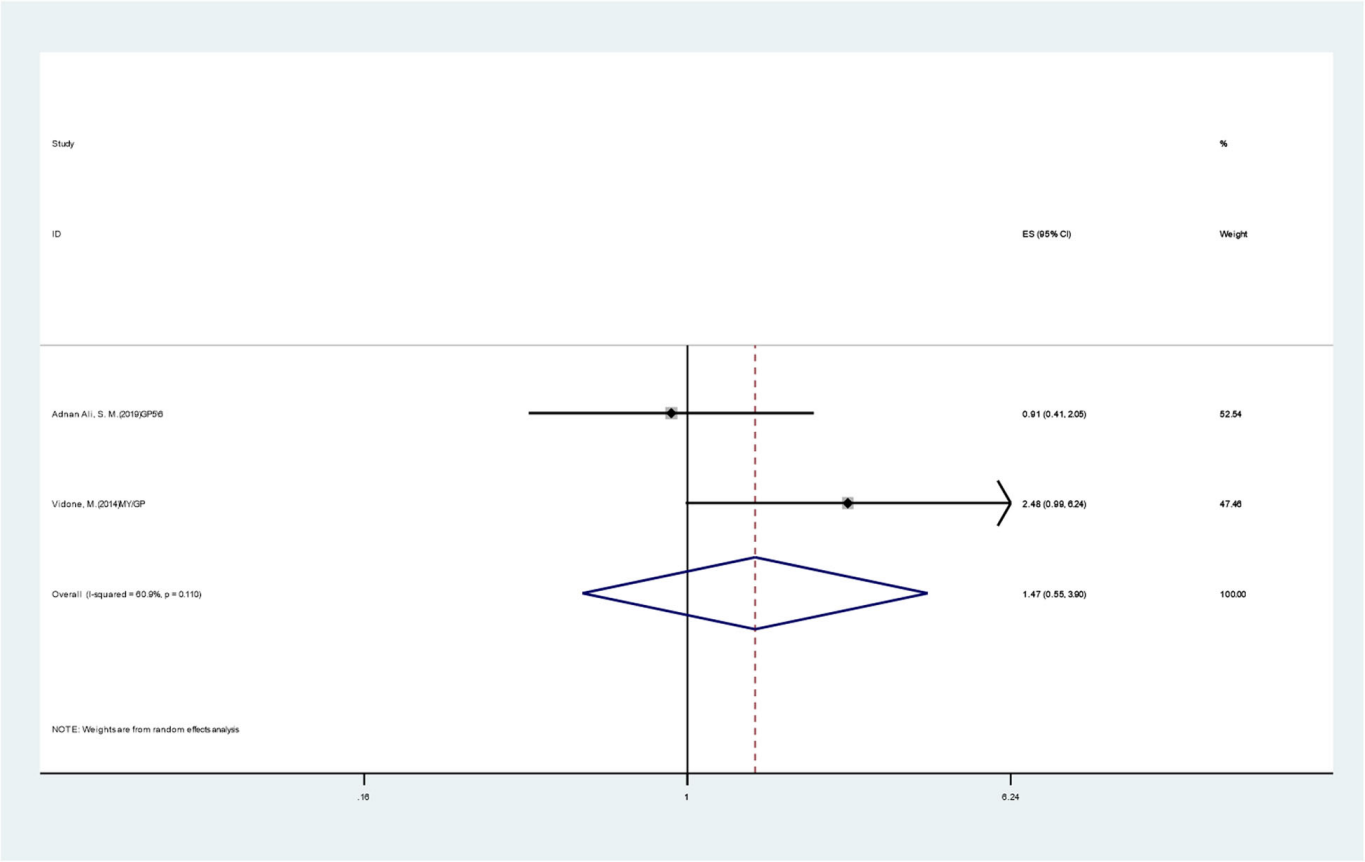
Pooled HR(OS) of HPV infection positive versus infection

The result of the I2 heterogeneity test was 60.9% (> 50%); thus, the random effect model was selected, and the final pooled HR was 1.467 (CI: 0.552–3.901), with a P value of 0.443, which was not statistically significant. HPV infection was not considered to affect tumor prognosis [12]. The results of the combination of individual indicators for each study are shown in Fig. 4.

#### HHV-6

In 2009, Crawford, J. R et al. conducted two studies among adults and children. In the study on adults, the HR of the late-indicator protein gp116/64/54 extracted from the M-H curve was 1.07 (CI: 0.76–1.5), and the P value was 0.6983. The HR of the early-indicator protein p41 extracted from the M-H curve was 1.25 (CI: 0.8–1.95), and the P value was 0.3271. Neither result was statistically significant. However, this is the same as the relationship between the early- and late-indicator proteins in the study of HCMV, and early-indicator proteins may be more inclined to indicate poor prognosis. In the study on children, it was difficult for researchers to extract the accurate HR and the OR for a survival period of 18 months from the M-H curve. Therefore, only the median survival time was recorded for prognostic evaluation. When gp116/65/54 was used as the detection index in children, the median survival time of the positive patients was 3.08 years (0.6–15.9), and that of negative patients was 3.25 years (0.1–10.9), with a P value of 0.245, showing no statistical difference. The median time of tumor progression was 2.67 years (0.42–7.1) for positive patients, and 2.25 years (0.1–9.3) for negative patients; the P value was 0.653, showing no statistical difference. Both studies showed that HHV-6 could not significantly affect the prognosis of patients.

#### Other viruses

In the study by Wang, Z. et al., in low-grade glioma patients, the HR of tumor cell infection with SV40 was 2.935 (CI: 1.4895–5.7843), and the P value was 0.0019, which was statistically different from that of non-infected patients. It was considered that the infection of patients' glioma cells with SV40 had a significant impact on the OS of low-grade glioma patients. Similarly, in patients with low-grade glioma, the HR of patients with WMSV-infected tumor cells was 2.109 (CI: 1.0663–4.1719), and the P value was 0.0019, showing a statistical difference. In the cohort of 308 glioma patients, the K113 risk ratio for infection versus non-infection was also not statistically significant because the *P* value was 0.6216. Because of the technical problem of image processing, we did not further extract the HR value, but only directly input the *P* value of cox analysis. It is believed that the infection of patients' glioma cells with SV40, WMSV or K113 has a significant impact on the OS of low-grade glioma patients.

## Discussion

The topic of viral infection and glioma has always been controversial. Since the study by Cobbs et al. first detected CMV-infected glial cells in glioma patients' tumor cells in 2002 [15], several laboratories have claimed to have detected the same results [16–18], but some have said that the results were negative [19]. In 2011, at a meeting on neuro-oncology, several scientists reached a consensus that glioma cells contain CMV [20]. However, several studies have been published since 2011 with mixed results [21–23]. The researchers with the positive results thought that the other studies lacked sensitivity. However, Cobbs et al. proposed normative standards for the process of detecting CMV in glioma [24]. In addition, in a randomized, double-blind study using ganciclovir, patients who received ganciclovir for more than 6 months had a better prognosis than those who received ganciclovir for less than 6 months [25]. OS at 4 years was 27.3% in Val>6M patients versus 5.9% in controls (p=0.0466). This trial also appears to support the idea of CMV infection in glioma patients, with reference to ganciclovir's antiviral profile [26]. However, in our meta-analysis, the pooled results showed that there was no statistically significant difference between the prognoses of patients with CMV expression and those without CMV expression, which could not provide a potential evidence for the existence of CMV infection in glioma cells. Alternatively, viral infection is not a strong factor in glioma formation or progression. Viral infection is more likely to be secondary to the patient's debilitated or immunosuppressed state. This result may be due to differences in detection sensitivity and specificity between studies. In some studies, extremely sensitive detection methods such as qPCR were used, which made the experimental results subject to artifacts or inaccurate due to contamination. Other viruses are less well studied and present a similar paradox [27, 28].

Our study had the following limitations: patients in different studies came from different geographical sources. The sensitivity or specificity of the detection methods and detection indexes were different among different studies. Standardized and unified detection standards can improve the accuracy of prognosis analysis. In one study, different models of PCR machines and different detection indicators were compared [29]. UL83 detection by qPCR and UL55 detection by nPCR showed the highest sensitivity. This suggested that the choice of the PCR technique and tumor samples to be used is important for the successful detection of low levels of the virus. The HR value obtained was derived from the univariate analysis. Compared with the HR values obtained by the multivariate analysis directly reported in other articles, the accuracy of the prognostic diagnosis was lower. With regards to studies on HPV and prognosis, the abstract of one study mentioned the prognosis analysis, but the researcher could not access the full text; excluding the study may have an impact on the integration of HR results. The other literature on the degree of CMV infection and the prognosis of patients was a meeting record, which was excluded based on our strict exclusion criteria.

Considering the homogeneity of the detection data, we only used the data from glioma tissue samples in this study. But viral infections may not only work by infecting glioma cells. Viruses in blood and cerebrospinal fluid, especially CSF, may affect the microenvironment of tumors, thereby affecting tumor progression and prognosis.

## Conclusion

Although this indicator of the IEA has shown predictive potential in studies on CMV infection degree and prognosis of glioma patients, SV and WMSV infections of low-grade glioma patients have a significant impact on the prognosis; the impact of virus infection on the prognosis of patients should still be carefully evaluated. From the current data, routine virus detection in tumor specimens should not be recommended for clinical use when current pathological and genetic tests have shown great ability to evaluate the prognosis of glioma. A larger number of prospective studies with uniform detection methods should be conducted in the future to verify the effect of viral infection on the prognosis of patients with glioma.

Another Potential purpose of this study was to indirectly demonstrate the presence of these viruses in GBM cells by means of prognostic analysis, if these viruses can play a role in promoting tumor progression. At least, we cannot easily conclude that these viruses, which are latent in tumor cells, can have a significant effect on the prognosis of patients. Virus infection may be nonspecific and unrelated to the tumor. Alternatively, viral infection may not be enough to dominate tumor progression. Anyway, more evidence is needed to establish a strong link between the virus and glioma.

## Data Availability

All articles were retrieved in Pubmed, Embase and Cochrane.Before data extraction, the articles underwent strict screening. Scientific and reliable methods are adopted to extract data. It can guarantee the data quality of the article and the validity of the material.

## Abbreviations

GBM: glioblastoma
LGG: low grade glioma
OS: overall survival
PFS: progression-free survival
CI: confidence interval
CMV: cytomegalovirus
HPV: human papillomavirus
HHV-6: human herpesvirus-6
SV40: simian virus 40
WMSV: woolly monkey sarcoma virus
HERV-K113: human endogenous retrovirus K113
HR: Hazard Ratio
PCR: polymerase chain reaction
qPCR: quantitative polymerase chain reaction
nPCR: nested polymerase chain reaction
IF: immunofluorescence
gB: glycoprotein B
gH: glycoprotein H
IEA: immediate early antigen
